# Thyroid Hormones and Functional Ovarian Reserve: Systemic vs. Peripheral Dysfunctions

**DOI:** 10.3390/jcm9061679

**Published:** 2020-06-01

**Authors:** Marco Colella, Danila Cuomo, Antonia Giacco, Massimo Mallardo, Mario De Felice, Concetta Ambrosino

**Affiliations:** 1 Department of Science and Technology, University of Sannio, via De Sanctis, 82100 Benevento, Italy; marco.colella@unisannio.it (M.C.); antonia.giacco@unisannio.it (A.G.); 2 IRGS, Biogem-Scarl, Via Camporeale, Ariano Irpino, 83031 Avellino, Italy; 3 Department of Molecular and Cellular Medicine, College of Medicine, Texas A&M University, College Station, TX 77843, USA; danila@tamu.edu; 4 Molecular Medicine and Medical Biotechnologies, University of Naples “Federico II”, 80131 Naples, Italy; massimo.mallardo@unina.it; 5 IEOS-CNR, Via Pansini 6, 80131 Naples, Italy

**Keywords:** thyroid hormones (THs), thyroid-stimulating hormone (TSH), thyroid hormone signalling, ovarian ageing, ovarian follicle, functional ovarian reserve (FOR), anti-Müllerian hormone (AMH)

## Abstract

Thyroid hormones (THs) exert pleiotropic effects in different mammalian organs, including gonads. Genetic and non-genetic factors, such as ageing and environmental stressors (e.g., low-iodine intake, exposure to endocrine disruptors, etc.), can alter T_4_/T_3_ synthesis by the thyroid. In any case, peripheral T_3,_ controlled by tissue-specific enzymes (deiodinases), receptors and transporters, ensures organ homeostasis. Conflicting reports suggest that both hypothyroidism and hyperthyroidism, assessed by mean of circulating T_4_, T_3_ and Thyroid-Stimulating Hormone (TSH), could affect the functionality of the ovarian reserve determining infertility. The relationship between ovarian T_3_ level and functional ovarian reserve (FOR) is poorly understood despite that the modifications of local T_3_ metabolism and signalling have been associated with dysfunctions of several organs. Here, we will summarize the current knowledge on the role of TH signalling and its crosstalk with other pathways in controlling the physiological and premature ovarian ageing and, finally, in preserving FOR. We will consider separately the reports describing the effects of circulating and local THs on the ovarian health to elucidate their role in ovarian dysfunctions.

## 1. Introduction

The accelerated decline in fertility and the onset of early menopause have been associated with loss of functional ovarian reserve (FOR), resulting in premature ovarian ageing. This decline is often asymptomatic and the underlying mechanisms are still poorly understood. Genetic and environmental factors contribute to this phenomenon. Both factors influence the number of the follicles, established in early life, and the hormonal assets required for their preservation and maturation during reproductive age [1].

Although conflicting, some epidemiological studies suggest a significantly higher prevalence of hypothyroidism, both overt and subclinical, in women with a genetic cause of diminished ovarian reserve (DOR) [2]. 

Thyroid hormones (THs) are involved in the normal growth, development and functions of many organs, including gonads. Their circulating levels are tightly regulated by feedback mechanisms active along the hypothalamic-pituitary-thyroid (HPT)-axis. Furthermore, cells and tissues can locally customize the TH signalling by regulating the life stage-specific expression of iodothyronine deiodinases (DIOs, enzymes involved in TH metabolism), TH transporters and, lastly, TH receptors (TRs). Their local modulation represents an additional and/or an alternative mechanism to maintain the peripheral T_3_ quota required for physiological processes, independently from fluctuations in circulating levels of THs [3]. Therefore, the organ/tissue-specific TH signalling is the result of thyroid hormones synthesis and of their peripheral metabolism. 

Our recent analyses of molecular mechanisms underlying both physiological and premature ovarian ageing revealed the impairment of several cellular functions controlled by TH signalling [4,5]. This review was conceived after these findings, when we searched PubMed using the combined terms “thyroid hormone metabolism” and “ovarian reserve” retrieving only 46 suitable articles. None article was retrieved when the terms “thyroid hormone signalling” and “ovarian reserve” were used. Both results were indicative of the poor characterization of the peripheral regulation of TH signalling in the ovary. 

Here, we will summarize results regarding the role of TH signalling in ovarian development, health and disease focusing on the specific activity of circulating THs and their peripheral metabolism/signalling in regulating FOR and ovarian health.

## 2. HPT and Peripheral Regulation of TH Metabolism during Ageing

**Figure 1:**
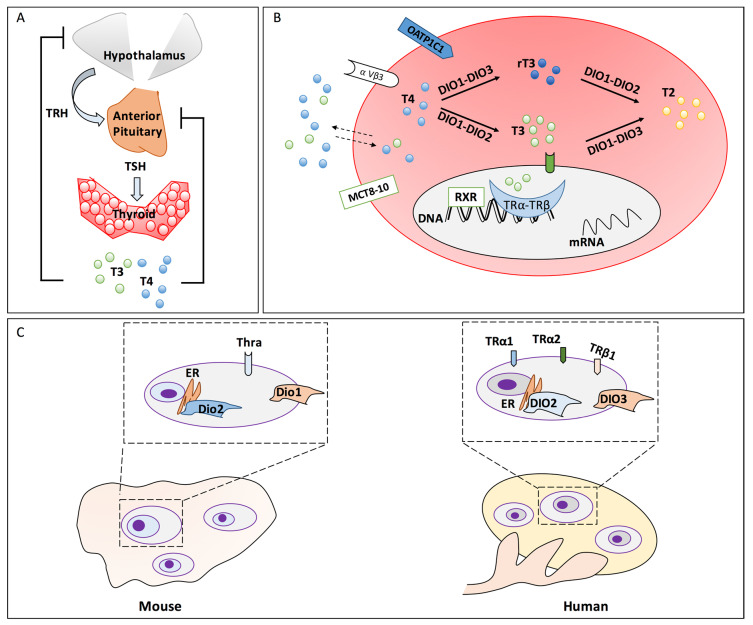
HPT-axis and peripheral TH metabolism/signalling in mammals. (**A**) Hypothalamic-Pituitary-Thyroid (HPT)-axis and its regulatory feedback loops. (**B**) Mechanisms/enzymes and other proteins involved in the cell/tissue-specific TH metabolism and signalling. (**C**) Description of the species-specific pattern of the ensemble of proteins involved in TH metabolism and signalling in the ovary. The ovarian follicles are evidenced in the dashed rectangles. The zoom on a single follicle is reported in the upper dashed box in order to evidence the ensemble of the expressed proteins involved in peripheral TH metabolism and signalling. Abbreviations: TRH, thyrotropin releasing hormone; TSH, thyroid-stimulating hormone; T4, thyroxine; T3, triiodothyronine; T2, 3,5-diiodo-L-thyronine; rT3, reverse triiodothyronine; DIO1, DIO2, DIO3, deiodinases; MCT8–10, monocarboxylate transporter 8–10; OATP1C1, organic anion transporting polypeptide 1C1; αVβ3, integrin alpha (V) beta (3); TRα and TRβ, thyroid nuclear receptors isoform α, β; RXR, retinoic acid X receptor; ER, endoplasmic reticulum; Thra, thyroid hormone receptor alpha (Mouse); TRα1, TRα2, TRβ1 thyroid hormone receptors isoform α1, α2, β1 (Human).

The synthesis and release of THs (T_4_ and T_3_) are tightly regulated by conserved mechanisms in vertebrates. Circulating T_4_ is controlled by a negative feedback mechanism involving the hypothalamus, the pituitary and the thyroid (HPT)-axis [6]. Specifically, the pituitary secretes the thyroid-stimulating hormone (TSH) that controls the synthesis and secretion of T_4_ and T_3_ by the thyroid. Both regulate in turn TSH release as well as the hypothalamic thyrotropin-releasing hormone (TRH) [7]. Conversely, intra-organ conversion of T_4_ to T_3_ provides negative feedback on the pituitary and on the hypothalamus inhibiting the TRH and TSH secretion, respectively (Figure 1A) [8,9,10].

The thyroid gland releases mainly the pro-hormone T_4_ and to a lesser extent T_3_, the biologically more active form of THs. Their synthesis requires the activity of a complex network of thyroid-specific enzymes. Briefly, iodine entry in thyroid follicular cells is mediated by two glycoproteins: sodium-iodide symporter (NIS) and pendrin [11,12]. The iodine oxidation is mediated by thyroid peroxidase (TPO) and, finally, it is incorporated into the thyroglobulin (TG) by a multistep process leading to the formation of T_4_. The prohormone T_4_ is converted to bioactive T_3_ (or to inactive rT_3_) in the thyroid and, mainly, in peripheral tissues by the deiodinases (Dio1, Dio2 and Dio3) [13,14,15]. Dio2 and Dio1 are T_4_/T_3_ activating enzymes and cooperate to maintain THs homeostasis, due to their differential expression in response to THs availability. Noteworthy, Dio2 regulates intracellular T_3_ and increases in hypothyroid subjects [16], whereas Dio1 regulates mainly circulating THs [17]. However, Dio1 can also inactivate T_4_ to rT_3_ and the sulfonated THs participating in the defence mechanism developed against iodide deficiency typical of hyperthyroidism [18]. Dio3 inactivates both T_4_ and T_3_ [19]. Moreover, both T_3_ and rT_3_ can be further metabolised to diiodothyronines (T_2_s), which also exhibits interesting metabolic activities (Figure 1B) [20,21,22]. Circulating THs are mostly bound to plasma proteins, such as thyroxine-binding globulin (TBG), whose level might influence TH signalling [23]. THs can be quickly liberated for entry into cells either by diffusion or by specific carrier-mediated mechanisms (e.g., OATP1C1, MCT8–10, etc.) [24,25,26].

The tissue specific TH signalling depends on the cellular content of TH receptors, which comprise the nuclear receptors (TRs), and the membrane receptors (e.g., αVβ3) [27,28,29,30]. Nuclear TRs act as transcription regulators in concert with other nuclear receptors, such as retinoic acid X receptor (RXR), for the recruitment of co-activators or repressors [31]. In mammals, four isoforms have been identified: TRα1, TRα2, TRβ1, and TRβ2 expressed in a tissue-specific manner [32,33,34]. On the other hand, integrin αVβ3 mediates THs non-genomic effects. Genomic and non-genomic pathways cooperate to determine the cellular-specific response to the TH signalling [35,36].

The regulatory role of peripheral TH signalling has been primarily described in metabolic processes, also becoming less efficient with ageing. Available data indicates that THs metabolism is impaired in aged organs. For instance, a reduction of Dio1 activity has been reported in thyroid and liver during ageing whereas age-related changes in TH receptors and transporters have been described in liver and kidney [37]. Circulating THs and TH signalling in peripheral organs were both reduced in a mouse model of progeria, in which the tissue-specific regulation of the activity of the deiodinases contributed to protect metabolic activity during ageing [38]. Although there is no definitive indication of the role of peripheral TH signalling in ovarian ageing, it is strongly evoked by the growing body evidence of its role in differentiation, proliferation and apoptosis in many organs, including the ovaries [39]. Indeed, in vitro and in vivo studies showed that Dio1 activity rose in the pituitary during ageing in order to maintain the local level of T_3_ [40,41], necessary to control the increase of circulating level of TSH [42]. Noteworthy, levels of TSH < 3.0 μIU/mL in euthyroid infertile patients have been associated with higher anti-Müllerian hormone (AMH) levels, a superior marker of FOR [43].

Taken together, the above-reported observations imply that the levels of circulating THs might not provide a sensitive and quantitative indicator of peripheral TH signalling in the ovaries, as well as in other organs, and that its deregulation could correlate with a premature loss of FOR [44,45].

## 3. TH and Other Pathways Involved in Preservation of FOR and Ovarian Health

Hormones, including THs, control various aspects of ageing [46]. The female reproductive system ages faster than the rest of the body: the ovaries are considered aged by the time a woman reaches the age of 45–50 years. Ovarian ageing is characterized by the progressive and silent decline of FOR, both in terms of quantity and quality of the oocytes. The menopause is the final step of this process. 

Genetic and environmental factors may contribute to the premature decline of FOR resulting in the Premature Ovarian Insufficiency (POI) [5]. Mutations in the genes of the TGF-beta family, such as *GDF9* and *BMP15* and *INHA*, have been associated with POI [47,48]. Specifically, GDF9 and BMP15 are produced by the oocytes whereas INHA is secreted by granulosa cells (GCs), they are collectively involved in the physiological maintenance of FOR [49,50,51]. AMH is another GC-specific member of this family playing an important role in ovarian ageing enough to be used as a marker of FOR. Notably, studies conducted in primary mouse GCs and in a human GCs cancerous cell line (KGN cells) evidenced that GDF9 and BMP15 could directly modulate AMH expression [52].

**Figure 2:**
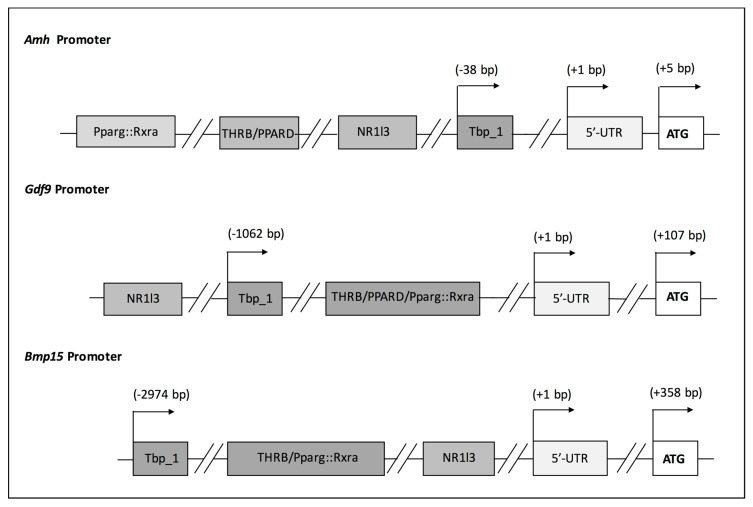
Analysis of mouse *Amh*, *Gdf9* and *Bmp15* promoters. List of transcription factor binding sites that were identified by the Jaspar tool analysing the 3000 bp upstream sequence of the genes. The ENSEMBLE Transcript ID were: (***Amh***) ENSMUST00000036016.5; (***Gdf9***) ENSMUST00000018382.6; (***Bmp15***) ENSMUST00000024049.7.

Different environmental factors (e.g., lifestyle, diet, exposure to environmental stressors) modulate AMH expression. It has been reported that the main metabolite of methoxychlor, a chlorinated hydrocarbon pesticide, increases AMH expression in rat immature GCs as well as in vivo [53]. On the contrary, other compounds such as dibutyl phthalate did not regulate its expression in cultured rat primary GCs [54]. Accordingly, we have also reported that environmental factors, i.e., ethylene thiourea (ETU) and different diets, could alter FOR whose status was assessed investigating the expression of the aforementioned genes. Specifically, *Amh* mRNA was considerably reduced in mice exposed to high- dose ETU (10 mg/kg/die) along with other transcripts whose inhibition was associated with physiological ovarian ageing. In the same experimental setting, we observed the concomitant substantial decrease in circulating T_4_ [5]. We assume that the hypothyroidism might be involved in POI onset participating in the transcriptional regulation of these genes. Indeed, we conducted the analysis of the mouse promoter of *Amh*, *Gdf9* and *Bmp15* genes in order to verify the prediction of thyroid hormone receptor binding elements (TREs). The results, schematized in Figure 2, evidence TREs in all of them. Similar results have been obtained also with their fish and human orthologs. Considering that conserved cis-regulatory elements regulate complex gene networks tuning basic developmental processes, such as establishment and maintenance of FOR, this points out the role of TH signalling in FOR establishment and preservation [55].

In zebrafish, the role of TH signalling in egg production has been investigated in females exposed to propylthiouracil (PTU) for 21 days. Exposed females presented the expected reduction of T_4_/T_3_ and an increased egg production together with a reduced size of the mature oocytes [56]. Recent studies on the reproductive seasonality in birds have also revealed that normal levels of circulating THs and their peripheral signalling are crucial to the normal development/lifespan of ovarian follicles. Specifically, in laying hens it was reported that the hyperthyroid status, induced by T_3_ administration, caused atresia of pre-ovulatory follicles and stoppage of laying eggs as well as the impaired synthesis of hormones in ovarian follicles at various stages of development in vitro. [57]. 

Furthermore, the role of TH signalling in mouse ovarian ageing could be evinced by a previous gene expression profiling analysis conducted in ovaries from young- and middle-aged mice in our laboratory [4,5]. Although the TH signalling was not directly highlighted by the bioinformatic analysis, we retrieved the reduced expression of a canonical TH-responsive gene (*Thrsp*, also known as *Spot14*) in the aged ovaries [5]. Since the mitochondria are well-characterised subcellular targets of THs [58], the inhibition of the oxidative phosphorylation further corroborates the possible reduction of TH signalling in ovarian ageing. Therefore, we have investigated a potential connection between the canonical pathways, identified by IPA analysis of the transcriptomic data [4,5], and TH signalling by reviewing the literature. As evidenced in Table 1, THs modulate the first nine identified canonical pathways evidenced in our analysis. Although not surprising, this is the first piece of evidence connecting the inhibition of ovarian TH signalling to physiological ovarian ageing in mice.

The role of the local TH metabolism and signalling in gonadal differentiation has been explored in mammals, especially in rodents and humans (Figure 1C). TH transporters (*slc16a2*, *slc16a10* and *slco1c1*), the deiodinases (*dio1*, *dio2*, *dio3a*, *dio3b*) and TH receptors (*thra* and *thrb*) have been reported to modulate Zebrafish (*Danio rerio*) development, however, their role has not been specifically investigated in the ovaries [59].

Contrarily, their ensemble has been evaluated in rodent gonads, especially in testis. Recently reviewed data from ENCODE Consortium have evidenced *Thra* as the most abundant TH receptor in rodent ovary, and *Mct8* (*Slc16a2*), *Lat1* (*Slc7a5*) and *Lat2* (*Slc7a8*) as the most expressed transporters. Regarding the deiodinases, the available data showed that *Dio2* is more expressed than *Dio1* whereas there are no data for *Dio3* (Figure 1C). Since expressing the ensemble of transporters, enzymes and receptors involved in the peripheral TH signalling, rodents have been pivotal in unravelling the mechanisms regulating TH availability and activity in the development of ovarian dysfunctions [60]. 

In humans, the mRNA and protein levels of the ensemble of TH-transporters, receptors and deiodinases have been reported in the different cellular components of the follicles and at different their maturation stages. Precisely, TRα1, TRα2 and TRβ1 were expressed in human ovarian surface epithelium and in oocytes of primordial, primary and secondary follicles. Both receptors were faintly detected in GCs of secondary follicles whereas they were clearly detected in GCs of antral follicles (Figure 1C). Lastly, *DIO2* and *DIO3* transcripts were found in both mature GCs and mature (MII) oocytes [61]. Moreover, recent findings underline the TSH- and TH-signalling cooperation in ovaries in in vivo and in vitro settings [62,63,64,65,66,67].

Taken together, the data suggest that circulating THs as well as local T_3_ signalling may contribute to the regulation of ovarian function.

## 4. Circulating TH/TSH Levels and Premature Ovarian Dysfunctions

Effects of different concentrations of T_3_ on ovarian function have been investigated in various in vitro systems. It was reported that T_3_ exposure promoted (FSH)-induced pre-antral follicle growth in vitro, by activation of the Akt pathway. The last-mentioned pathway plays a crucial role as an anti-apoptotic factor for the GCs in rat [68], as also evidenced by the gene expression profiling study conducted in our laboratory (Table 1). Additionally, this observation was confirmed in a study evaluating T_3_ protective role in rat GCs exposed to a chemotherapeutic drug [69]. Besides, it has been shown the presence of TSH-receptor in human GCs and the increase of cAMP upon TSH stimulation [61].

HPT-axis is physiologically related to the hypothalamic-pituitary-gonads (HPG)-axis, both regulate reproductive functions [70]. As said, the zebrafish thyroid is comparable to the mammalian one in terms of genes responsible for thyroid development and/or for TSH function [71,72]. It has been shown that the hyperthyroidism in zebrafish larvae inhibited the aromatase (*cyp19a1*) activity, leading skewed sex ratio in favour of males [73]. Furthermore, it has been reported that adult females exposed to PTU showed the expected reduction of T_4_ and T_3_ and the increase of the steroidogenic transcripts (*star*, *hsd3b* and *hsd17b*) after short or long exposure. Supposedly, elevated levels of FSH and LH caused their altered expression [74]. Despite that, the role of thyroid hormones in regulating FOR in zebrafish is far from being defined. 

Recently, the association of hypothyroidism with impairment of FOR has been examined in mice and rats after administration of PTU and low-iodine diet, respectively [75,76]. In both cases, the number of primordial, primary and preantral follicles was reduced whereas none significant change of atretic follicles was reported. Although none explanation was supplied, the data suggested that the numeric reduction of preantral and antral follicles was not due to their degeneration under hypothyroid condition [76,77]. Noteworthy, in a previous paper, the same authors reported an impairment of FSH and LH surge with a concomitant alteration of the antioxidant enzymes (e.g., catalase, SOD1, and NOS) in ovaries from hypothyroid rats [78]. Experimental studies conducted in Wistar rats demonstrated that hyperthyroidism increased the number of secondary and tertiary follicles whereas reduced the follicular atresia [79]. The effects of hyperthyroidism have been investigated in several reports. In prepuberal and adult rats T_3_ treatment altered the ovarian steroidogenesis suggested as the cause of the impaired folliculogenesis and ovulation [80]. Furthermore, T_3_ cooperated with FSH to promote preantral follicle development in mice by increasing *Xiap* and by reducing *Bad* mRNA levels [81]. Contrasting results have been reported regarding the effect of L-thyroxine, used to treat hypothyroidism, on ovarian health in rats. Specifically, Jiang et al. reported that L-thyroxine treatment of spontaneously hypothyroid rdw rats improved follicular development, but did not restore the pre-ovulatory surge of LH [82], whereas Zheng et al. reported a reduced number of primordial and antral follicles [83]. Other studies have been conducted in rats treated with PTU to promote prepuberal hypothyroidism. The published data evidenced that PTU reduced the proliferation of GCs in follicle-stage dependent manner [84].

Lastly, it should be accounted that maternal thyroid dysfunction in rats, both hypothyroidism and hyperthyroidism, affects the ovarian development of the offspring by reducing the follicle number at different developmental stages [80]. Despite the establishment of the OR during the foetal and the neonatal life stages and the effects of maternal hypothyroidism on the ovarian health of the offspring, the connection between local THs and ovarian dysfunctions needs further investigation.

Some of these aspects have been investigated also in humans. Although known causes of POI, include radiation, chemotherapy, X chromosome deletions and defects in genes codifying for the gonadotropin hormones or receptors, about 90% of the cases remain idiopathic [85]. THs likely play a role in POI onset and progression because of their cross-talk with other hormonal pathways (e.g., oestrogen, prolactin, IGF−1 and GnRH) impairing the folliculogenesis. The prevalence of hypothyroidism ranges between the 0.3%–4.3% in adult women and it is often associated with the presence of thyroid antibodies (e.g., AbTPO, AbTG) [86]. Although debated, the link between increased infertility/ovarian dysfunction with hypothyroidism/thyroid antibodies has been explored, evidencing the association of high levels of thyroid antibodies and several reproductive dysfunctions, including POI [87,88,89,90,91]. Michlakis and co-authors showed an increase of thyroid diseases in women affected by DOR when compared to other patients whose infertility had other origins. Therefore, the screening for TH levels and thyroid antibodies is currently recommended in women suffering from POI with unknown aetiology [92]. More recently, two conflicting studies have investigated the association between the levels of thyroid antibodies and the reduction of the FOR. The first, published in 2015, is a retrospective study involving about 5000 women. Among them, about 1/10 were affected by a diminished ovarian reserve (DOR) and about the same number had a normal ovarian reserve. Both groups did not show statistically different concentrations of fT4, TSH and AbTPO antibodies. Higher prevalence of sub-clinical hypothyroidism or hypothyroidism was observed when DOR had exclusively a genetic cause [2]. The second report, published in 2019, describes a 12-year follow-up study aimed at assessing the modulation trend of THs and AbTPO antibodies in women. FOR was determined by measuring serum AMH concentration. Its first determination, considering the age-specific AMH reference values, was used to group the patients in quartiles: Q1, grouping the women with the lowest AMH level, up to Q4 including the ones with highest AMH level. In three different follow-up visits, as at the baseline, TSH, fT4 and AbTPO antibodies were also measured. Interestingly, none statistically relevant difference in circulating THs was detected at the baseline whereas the AbTPO antibodies concentration was higher in women included in Q1. A progressive decrease of fT4 and an increased level of AbTPO antibodies were detected in all the quartiles over time [93]. Accordingly, a previous study, involving about 1000 Chinese women, reported the increase of AbTPO antibodies concomitant with idiopathic DOR [94]. 

Although the prevalence of hyperthyroidism is lower than hypothyroidism (1.3%), about 5.8% of hyperthyroid women are infertile [95]. This may be due to the production of anti-TSH antibodies whose increase has been associated with primary and secondary infertility.

Given the above data, we suggest that circulating THs and TSH might contribute to the establishment and maintenance of FOR. 

## 5. Peripheral TH Metabolism/Signalling and Markers of Ovarian Reserve: Potential “Local” Crosstalk

As stated, the role of peripheral thyroid metabolism and signalling has been poorly explored in ovary both in vitro and in vivo. In vitro systems have been pivotal in suggesting the potential crosstalk between the different pathways involved in FOR preservation. They have been used to explore also the crosstalk between the gonadotropins and thyroid pathways, above all TSH, on gonadal development and vice versa. Indeed, the TH/TR complexes might exert their biological function interconnecting with other signalling pathways including AMH, GDF9, BMP15, IGF or other endocrine hormones (e.g., FSH, LH), playing a role in POI onset [96,97].

As said, these aspects have been analysed in vitro. The expression of GDF9, BMP15 and AMH during the developmental stages of the follicles is fundamental to the activation of signalling pathways directly involved in FOR preservation [52,98,99]. Despite ovarian cell lines carrying the deletion of one or more of genes of the ensemble of factors involved in cellular TH metabolism and signalling, the crosstalk of the above-reported pathways with intracellular T_3_ signalling has been explored in the ovaries [100,101]. Firstly, it was reported the involvement of T_3_ in the amplification of FSH-R signalling in the differentiation of porcine GCs, due to the increased transcription of the FSH receptor gene [102]. Subsequently, Tsang and co-authors confirmed the interaction of FSH and T_3_ signalling in increasing the FSH-R levels in rat pre-antral follicles via GDF9. It has also been shown that T_3_ and FSH co-treatment enhanced steroid biosynthesis driven by an increased expression of cytochrome P450 lanosterol 14α-demethylase (Cyp51), a mediator of T_3_- and FSH-induced follicular development [103]. These results indicated the potential role of TH and gonadotropin signalling crosstalk in the ovaries [104]. The crosstalk between the proteins of the TGF-beta family and THs has also been indirectly suggested in an in vitro study conducted in bovine cumulus cells stimulated with GDF9 and BMP15. After stimulation, cells showed an increase of a circular RNA hosted in TRAP80, a component of different multi-subunit complexes facilitating their function as a transcriptional factor, including TRs [105]. Although debatable, it is likely that elevated circulating TSH levels, often associated with overt or subclinical hypothyroidism, may be detrimental for FOR. For instance, the TSHR/IGF−1R cross-talk is an important mechanism for the regulation of cellular activity in thyrocytes as well as the expression of thyroid-specific genes and activation of MAPK pathways [106]. Although this crosstalk has not been explored yet in the ovary, we underscore the presence of both receptors in the tissue. Additionally, it has been reported that TSH-R expression is increased by the gonadotropin-driven cAMP cascade and inhibited by oestradiol production in cultures of rat follicles and primary GCs. Lastly, thyrostimulin, produced by the oocytes, is known to be TSH-R main activator in the ovary [107]. The crosstalk between the gonadotropins and thyroid pathways has been investigated in different animal models, including teleosts (goldfish) and mammals (rodents and humans) [104,108,109]. In goldfish, T_3_ inhibited the expression of LH in the pituitary [110]. Accordingly, the inhibition of circulating LH was evidenced in hyperthyroid rats [111]. Conversely, other reports have evidenced that LH mRNA was unaffected by hypothyroidism or T_3_ replacement in rat pituitary [112,113]. Finally, in hyperthyroid women higher levels of circulating LH have been reported, a sign of a paradoxical effect observed also in goldfish [70,114]. In the above-cited studies, the regulation of LH transcription, as well as FSH, was suggested as the mechanism by which THs modulated the steroidogenesis and the expression of the oestrogen receptors in the ovary [115]. Recently, this indication was corroborated by experiments conducted in zebrafish and mouse models carrying the homozygous deletion of the genes codifying for TH receptors and deiodinases.

A dio2 mutant was established in zebrafish, which showed a dramatic decrease of T_3_ level in the gonads leading to a male-biased development. It was reported that the permanent *dio2* deficiency determined severe fertility issues associated with a defect in egg laying [116]. The fertility and the ovarian phenotype were further investigated in wild type and mutant animals. The latter showed an increased expression of *dio1* and *thra* transcripts in the ovaries. In addition, dio2 mutant presented an increase in the primary oocytes and to a lesser extent of the vitellogenic ones along with the inhibition of the ovulation. This phenotype was considered the result of the suppression of the steroidogenesis. Indeed, the ovarian reduction of T_3_ signalling resulted in the inhibition of ovarian oestrogen levels and concomitant down-regulation of *hsd11b2* and upregulation on *esr2b* transcripts. This effect could be rescued by T_3_ supplementation [117].

In a rat model of iodine deficiency (LID), induced by a low-iodine diet, it was shown a slight reduction of Dio1 and about 2-fold increase of Dio2 activity, indicating a local compensatory mechanism [118]. Although not further characterised, similar effects were shown in zebrafish dio2 KO. It has also been reported that the reproduction was severely hampered in DIO3KO mouse. However, the effects on ovarian health have not been directly analysed [119]. On the contrary, none major effect on female fertility was described in DIO2KO and DIO1KO mice [120]. Several mouse models have been developed carrying a homozygous deletion in the TRs genes, even though never ovary-targeted. Nevertheless, some of these animal models showed fertility problems that were related to altered ovulation, as in the TRα2 knockout females [121].

Overall, the data evidence the importance of TH signalling in the preservation of FOR, as a result of its crosstalk with other signalling pathways strictly involved in ovarian health.

## 6. Conclusions

Taken together, the data evidence that abnormal levels of THs, especially during puberty and fertile age, might result in ovarian dysfunction throughout the entire life. Different mechanisms may contribute, ranging from the altered circulating THs levels and/or their peripheral metabolism/signalling to their crosstalk with signalling pathways pivotal for the preservation of FOR. Well documented studies indicate that thyroid dysfunctions, especially in early-life stages, may determine subfertility or infertility, menstrual/oestrous irregularity, anovulation.

**Figure 3:**
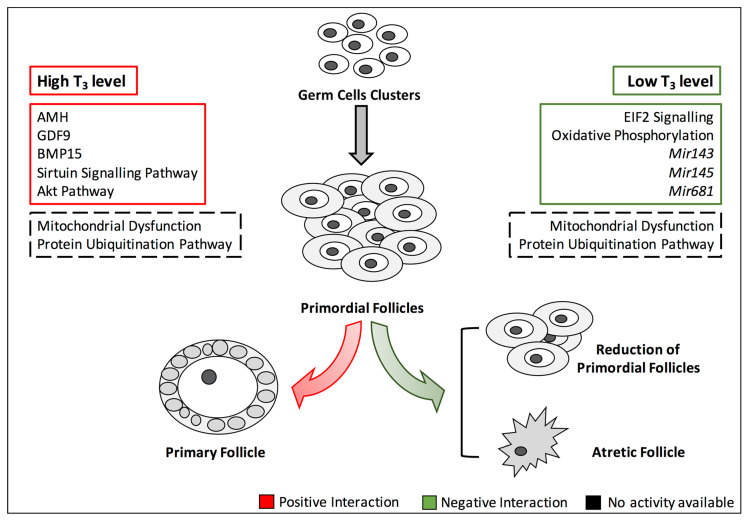
Signalling pathways playing a positive or negative role in FOR homeostasis. The figure depicts the main signalling pathways involved in FOR maintenance and how they are impaired by altered TH signalling. Green and red boxes indicate negative and positive interactions playing a role in FOR homeostasis, respectively. Black dashed boxes define “unknown” interaction type. The reported molecular functions are publicly available in Cuomo et al., 2018. Abbreviation: T_3_, triiodothyronine; AMH, anti-Müllerian hormone; GDF9, growth differentiation factor 9; BMP15, bone morphogenetic protein 15.

Here, we have reported results from retrospective studies of women with thyroid dysfunction as well as in vivo and in vitro studies conducted in animals and/or ovarian cell cultures models of hypothyroidism or hyperthyroidism. Although still debatable, the data suggest that sub-clinical and/or overt hypothyroidism reduces the number of growing follicles and increases follicular atresia. Moreover, the results of studies investigating the relationship between the hyperthyroidism and the ovarian health are conflicting. In fact, an increase of T_3_ among patients suffering from the polycystic ovary syndrome (PCOS) has been associated with enhanced activity of FSH, which preserves FOR [122]. These effects could be direct or an indirect consequence of the crosstalk with other signalling pathways playing either a positive or negative role in the preservation of FOR (Figure 3).

This review aims at shedding the light on the peripheral TH signalling involvement in the maintenance of FOR. Very little data have been published in this regard and the retrieved publications poorly addressed the molecular mechanisms underlying the role of local thyroid hormone metabolism/signalling in the ovaries. We suppose that this depends on the models adopted in the reviewed studies, in which TH signalling impairment results from the exposure to environmental stressors, i.e., low-iodine intake, endocrine disruptors, or by the generation of whole-body knockouts.

We believe that more effort is needed to develop adequate models to characterise the role of TH signalling in the ovary such as GCs, mouse and/or zebrafish carrying an ovarian specific deletion of the genes codifying the proteins regulating TH metabolism and signalling. They are essential in understanding the effects of THs impairment in the establishment and preservation of FOR. Their development will be pivotal in dissecting the molecular mechanisms of thyroid hormone action in regulating the FOR under physiological and disease-related conditions.

## Figures and Tables

**Table 1:** Canonical pathways, targeted by THs, affected in physiological ovarian ageing. IPA analysis of microarray data, previously published (Cuomo et al., 2018), mapped nine top biological processes altered during physiological ovarian ageing. They are listed together with the deregulated genes that contribute to their identification. The statistical relevance, the activation status, and the regulatory role of THs of the pathways are reported as −log(*p*-value), z-score, and citations, respectively.

Canonical Pathways Identified by Ingenuity Analysis (IPA)	Molecules (Genes) Identifying the Pathway	z-Score	−log(*p*-Value)	References Pointing out the Regulatory Role of THs Relative to the Pathway
EIF2 Signalling	*Eif2s2*, *Eif3a*, *Eif3e*, *Eif3m*, *Eif5B*, *Rpl11*, *Rpl13a*, *Rpl15*, *Rpl17*, *Rpl26*	−3.207	23.10	Torres_Manzo AP. et al.; Oxid Med Cell Longev 2018 Takahashi K. et al.; J Biol Chem. 2014 Goulart-Silva F. et al.; Thyroid 2012 Arrojo E Drigo R. et al; Molecular Endocrinology 2011
Regulation of eIF4 and p70S6K Signalling	*Eif2s2*, *Eif3a*, *Eif3e*, *Eif3m*, *Paip2*, *Ppp2r5a*, *Rps12*, *Rps18*, *Rps23*, *Rps24*	_	12.40	Ediriweera MK. et al.; Semin Cancer Biol. 2019 Manfredi GI. et al.; Endocrine 2015 Kenessey A. and Ojamaa K.; The Journal of Biological Chemistry 2006
Oxidative Phosphorylation	*Atp5mc2*, *Atp5mg*, *Atp5po*, *Cox17*, *Cox6a1*, *Cox7a2l*, *Ndufa1*, *Ndufa4*, *Ndufb1*, *Ndufb11*	−2.887	8.97	Lombardi A. et al.; Front Physiol. 2015 Harper ME. and Seifert EL.; Thyroid 2008 Weitzel JM. et al.; Exp Physiol. 2004 Martinez B. et al.; Journal of Neurochemistry 2001 Harper ME. et al.; Biochem Soc Trans. 1993
mTOR Signalling	*Eif3a*, *Eif3e*, *Eif3m*, *Ppp2r5a*, *Rps12*, *Rps18*, *Rps23*, *Rps24*, *Rps25*, *Rps27a*	_	8.42	Varela L. et al.; J Pathol. 2012 Kenessey A. and Ojamaa K.; The Journal of Biological Chemistry 2006 Cao X. et al.; Molecular Endocrinology 2005
Protein Ubiquitination Pathway	*Bag1*, *Hsp90aa1*, *Hspa9*, *Psma2*, *Psma 4*, *Psma 7*, *Psmb1*, *Psmb3*, *Psmb5*, *Psmb6*	_	7.71	Egri P. and Gereben B.; J Mol Endocrinol. 2014 Dace A. et al.; PNAS 2000
Mitochondrial Dysfunction	*Atp5mc2*, *Atp5mg*, *Atp5po*, *Cox17*, *Cox6a1*, *Cox7a2l*, *Gpx4, Ndufa1*, *Ndufa4*, *Ndufb1*	_	7.69	Tilly JL and Sinclair DA. Cell Metab. 2013 Harper ME. and Seifert EL.; Thyroid 2008 Venditti P. and Di Meo S.; Cell Mol Life Sci. 2006 Siciliano G. et al.; Mol Med. 2002Chen YD. and Hoch FL.; Arch Biochem Biophys. 1976
Sirtuin Signalling Pathway	*H3f3a/H3f3b*, *Ndufa1*, *Ndufa4*, *Ndufb1*, *Ndufb11*, *Ndufb9*, *Polr1d*, *Sdhb*, *Slc25a4*, *Tomm70*	0.378	3.10	Al-khaldi A. and Sultan S.; BMC Endocr Disord. 2019 Xiao-Ling Zhou et al.; J Ovarian Res. 2014 Suh J. et al.; PLoS One 2013 Akieda-Asai et al.; PLoS One 2010
TCA Cycle II (Eukaryotic)	*Dld, Mdh1, Sdhb*	_	2.76	Zhou J. et al.; Front Physiol. 2019 Mitchell CS. et al.; J Clin Invest. 2010
Mitotic Roles of Polo-Like Kinase	*Anapc13*, *Cdc16*, *Hsp90aa1*, *Ppp2r5a*	_	2.35	Wang K. et al.; J Biol Chem. 2014 Russo A.M et al.; Thyroid 2013
